# Effect of a theory-based coach education program on the objectively assessed motivational climate in grassroots football across four European countries: results from the Promoting Adolescent Physical Activity (PAPA) project

**DOI:** 10.3389/fpsyg.2026.1563931

**Published:** 2026-08-05

**Authors:** Yannis S. Tzioumakis, Damien Tessier, Nathan Smith, Priscila Fabra López, Charalambos Krommidas, Nikos Comoutos, Panagiotis Keramidas, Paul R. Appleton, Philippe Sarrazin, Isabel Balaguer, Nikos Digelidis, Eleanor Quested, Athanasios G. Papaioannou, Joan L. Duda

**Affiliations:** 1 Department of Physical Education and Sport Science, University of Thessaly, Trikala, Greece; 2 SENS, Université Grenoble Alpes, Grenoble, France; 3 University of Coventry, Coventry, United Kingdom; 4 Department of Psychology, Universidad Europea de Valencia, Valencia, Spain; 5 School of Sport and Exercise Sciences, Manchester Metropolitan University, Manchester, United Kingdom; 6 Institute of Sport, Manchester Metropolitan University, Manchester, United Kingdom; 7 Department of Social Psychology, Faculty of Psychology and Speech Therapy, University of Valencia, Valencia, Spain; 8 Curtin University, Perth, WA, Australia; 9 School of Sport, Exercise and Rehabilitation Sciences, University of Birmingham, Birmingham, United Kingdom

**Keywords:** Achievement Goal Theory, coach training, empowering coaching, motivational climate, observation, self-determination theory, youth football

## Abstract

**Introduction:**

This study examined the longitudinal effects of the *Empowering Coaching*™ program, designed to promote more adaptive motivational climates as created by coaches in grassroots youth football across different countries.

**Methods:**

Sixty-eight coaches from four countries (England, France, Spain, and Greece) were randomly assigned to either the intervention or control group. Coaches’ behaviors were assessed at three time points during the season using a validated observational system based on motivational theories.

**Results:**

A series of linear and quadratic multilevel models revealed that, compared to the control group, intervention coaches demonstrated a significant reduction in disempowering behaviors (i.e., ego-involving climate) across the season and a temporary increase (assessed 1 month post-intervention) in features of an empowering climate (i.e., autonomy support and relatedness support).

**Conclusion:**

Findings highlight the training program’s short- and long-term impacts and point to the need for continued support to sustain positive coaching practices and reduce the occurrence of negative coaching behaviors.

## 1 Introduction

Youth sport is one of the most widely practiced forms of organized physical activity worldwide, offering young people opportunities for social development, skill acquisition, and personal growth (Behzadnia et al., 2025). However, sport participation can also lead to negative experiences, such as burnout, anxiety, and dropout, especially when athletes are exposed to maladaptive coaching practices (Espinoza-Gutiérrez et al., 2024).

The coach is widely recognized as a key figure shaping young athletes’ experiences in sport. The concept of motivational climate in the sporting realm refers to the social psychological environment operating in training and competition (e.g., as created by coaches), which influences athletes’ motivational processes. The coach-created motivational climate is a function of what coaches say and do in terms of the provision of feedback, organization, and the nature and basis of recognition and evaluation provided (Duda and Balaguer, 2007).

The majority of the work on the concomitants of the motivational climate stems from Achievement Goal Theory (AGT; Ames, 1992; Nicholls, 1989) and Self-Determination Theory (SDT; Ryan and Deci, 2017). This research has centered on the nature and significance of particular features of the social-psychological environment surrounding sport participants, and the implications of these characteristics of the perceived motivational climate on athletes’ cognitions, affective responses, and behaviors (Duda and Balaguer, 2007). As such, interventions targeting coach behaviors grounded in these motivational theories can play a central role in promoting positive developmental outcomes in youth sport.

AGT has served as a framework for explaining motivational functions within the coaching environment and postulates that coaches can create a motivational environment that is more or less task- and ego-involving (Ames, 1992). A task-involving motivational climate is characterized by recognition of effort and self-improvement, the promotion of cooperation, and ensuring that each team member’s role is valued (Newton et al., 2000). Conversely, an ego-involving climate focuses on evaluating competence based on normative standards, encourages intra-team rivalry, places emphasis on outperforming others, and emphasizes mistake-contingent punishment (Newton et al., 2000). The motivational climate literature within youth sport suggests that a task-involving coaching climate is linked to a series of adaptive processes and consequences for athletes, such as increased intrinsic motivation (Amaro et al., 2023), lower anxiety levels (Smith et al., 2007), higher perceived competence, and overall athlete wellbeing (Fry et al., 2012; Reinboth and Duda, 2006). In contrast, an ego-involving coach-created motivational atmosphere has been linked with athletes’ elevated stress and anxiety levels as well as with increased negative affective responses (Lochbaum and Sisneros., 2024) and deceptive practices (Harwood, 2008).

SDT (Ryan and Deci, 2017; Vansteenkiste et al., 2020) complements AGT by postulating that, in addition to coach behaviors that influence athletes’ perceptions of competence (i.e., experiences of mastery and effectiveness), those that affect feelings of autonomy (i.e., experiences of being a causal agent) and relatedness (i.e., experiences of belonging and connection with others) also impact athletes’ motivation and ensuing outcomes. Past studies have emphasized environmental strategies that support or thwart the needs for autonomy, competence, and relatedness. An autonomy-supportive coach acknowledges feelings and perspectives, provides meaningful choices, offers rational encouragement, encourages initiative, provides non-controlling feedback, and facilitates athletes’ active involvement in interesting, meaningful, and engaging activities (Mageau and Vallerand, 2003; Reeve, 2009). In contrast, a controlling interpersonal style is characterized by overt personal control over athletes, devaluation of athletes’ perspectives, the controlling use of rewards or language, intimidation (i.e., coercive or seductive pressures and demands), and conditional regard (Bartholomew et al., 2010; Reeve, 2009). An interpersonal style that supports the need for competence is characterized by a coach who conveys the expected learning outcomes, provides athletes with clear instructions, and offers informative guidance as needed while the task is in progress, as well as constructive feedback after the task has been completed (Jang et al., 2010; Sierens et al., 2009). In contrast, a coach who provides unclear or inconsistent instructions, fails to monitor students’ understanding of the rules and requirements, and offers little guidance or feedback during gameplay may undermine athletes’ need for competence (Aelterman et al., 2019). A relatedness-supportive coaching environment is marked by positive coach–athlete interactions, which are characterized by emotional support, feelings of concern, closeness, respect, warmth, or caring (Fry and Gano-Overway, 2010), and unconditional acceptance and inclusion, communicated in a consistent, non-contingent manner. In contrast, a relatedness-thwarting coaching environment is characterized as cold and critical (Skinner and Edge, 2002). A recent meta-analysis showed strong positive associations between coach autonomy support behaviors and athlete outcomes, such as autonomous motivation and indicators of wellbeing (e.g., positive affect, subjective vitality, life satisfaction, and self-esteem) (Mossman et al., 2024).

**Figure 1:**
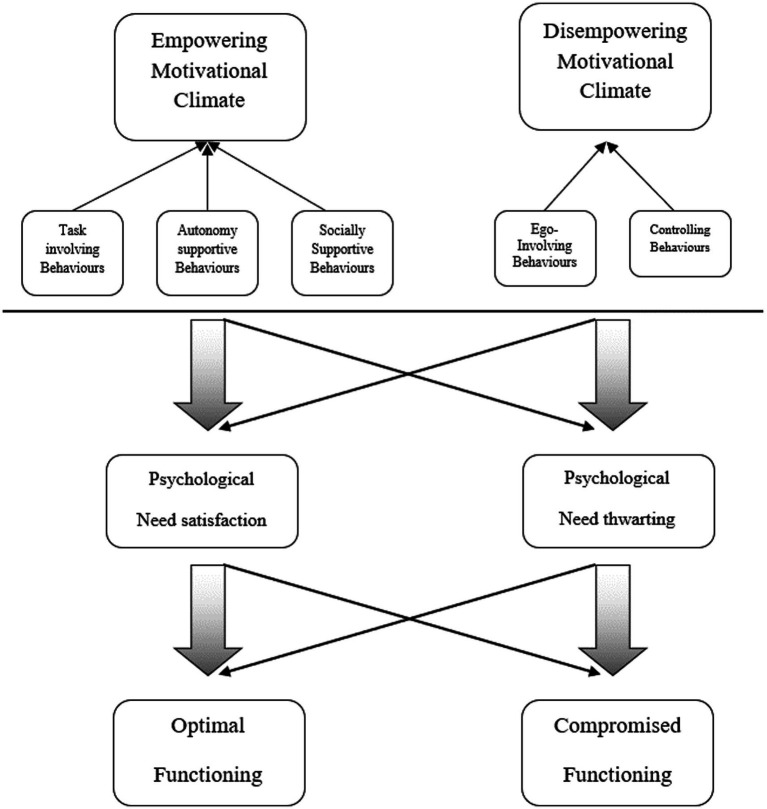
Duda's (2013) conceptualization of empowering and disempowering motivational climates incorporating consideration of AGT and SDT constructs.

More recent theoretical advances (Duda, 2013; Duda and Appleton, 2016; Duda et al., 2024) have proposed an integrated model of the motivational climate that considers constructs and predictions stemming from AGT and SDT frameworks, defining environments as “empowering” when they promote autonomy, task involvement, and relatedness, and “disempowering” when they are marked by controlling, ego-involving, and relatedness-thwarting behaviors. The present study was drawn from Duda’s integrated framework in regard to the intervention implemented, which aimed to optimize the coach-created motivational climate, as well as to examine how differences and changes in the motivational climate were assessed (Figure 1).

The vast majority of AGT- and SDT-based research on the motivational climate in sport (as well as studies grounded in the integrated framework) has relied heavily on self-report measures. However, questionnaire methodology suffers from inherent shortcomings such as potentially biased perceptions of the motivational climate (Murayama et al., 2012) and possible common-method variance (De Meyer et al., 2014). To overcome these limitations and given the underpinning conceptual framework in the present study, the Multidimensional Motivational Climate Observation System (MMCOS; Smith et al., 2015) was employed to objectively capture coaching behaviors aligned with the theoretically integrated model proposed by Duda and colleagues (Duda, 2013; Duda and Appleton, 2016; Duda et al., 2024).

Despite the theoretical promise of integrated models (Duda, 2013; Duda et al., 2024) of the motivational climate to enhance coaching practice, their empirical testing through large-scale, cross-national interventions is limited. Additionally, many existing intervention studies of the coach-created motivational climate have not adequately reported the sustainability of behavior change among coaches over time. This is especially relevant given evidence that without ongoing support, initial gains from training programs may fade (Cheon et al., 2015).

The present study aimed to address these gaps by evaluating the effects of the *Empowering Coaching*™ coach education program (Duda et al., 2013) on the coach-created motivational climate, delivered as part of the European Promoting Adolescent Physical Activity (PAPA) project. Using the MMCOS, we assessed whether the intervention improved the observed motivational climate created during training sessions across grassroots football teams in four countries (England, France, Greece, and Spain). Specifically, we hypothesized that:

1. Coaches in the intervention group would be observed to exhibit behaviors reflecting higher levels of empowering climate features (overall and regarding autonomy support, task-involving climate, and relatedness support sub-dimensions) and lower levels of disempowering features (overall, and regarding controlling behavior, ego-involving climate, and relatedness-thwarting sub-dimensions) compared to those in the control group coaches.
2. These effects would be evident in both short-term (1 month) and long-term (season-end) observations.

## 2 Method

### 2.1 Participants

Sixty-eight (66 male and two female participants) grassroots football coaches (*M_age_* = 35.7, SD = 9.7; *M_coaching experience_* = 6.89, SD = 4.4) of athletes aged 10 to 15 years (*M_team age_* = 11.92, SD = 1.45) from England (*n* = 17), France (*n* = 11), Spain (*n* = 18), and Greece (*n =* 22) were recruited from the coach participant pool within the PAPA project. The coaches were randomly allocated to an intervention arm (36 coaches; *M_age_* = 35.2, SD = 9.3; *M_coaching experience_* = 6.71, SD = 3.7) or a control condition (32 coaches; *M_age_* = 36.1, SD = 10.1; *M_coaching experience_* = 7.20, SD = 4.9). The former group received the targeted coach education training.

Ethical approval for this study was granted by the corresponding ethics boards of the co-investigators’ universities. Participants gave informed consent to be filmed during training sessions at three time points (T1–T3). Soccer players’ parents/guardians were also informed about the filming and the overarching purpose of the study (to observe coaches leading training sessions across a season) and were allowed to opt their child out of the research. Moreover, the young players were also informed that they could withdraw from the study (i.e., not participate in the session to be filmed) despite consent being provided by their guardians.

Limited coach attrition occurred across the three time points (*n* = 9). Attrition that did occur was due to various reasons (e.g., team change, club change, and profession change). In Time 2 (T2; 1-month follow-up), data were obtained from 66 coaches (England, *n* = 17; France, *n* = 11; Spain, *n* = 16; and Greece, *n =* 22). In Time 3 (T3; 6-month follow-up), 59 coaches were filmed (England, *n* = 13; France, *n* = 8; Spain, *n* = 16; and Greece, *n =* 22).

### 2.2 Procedure

**Figure 2:**
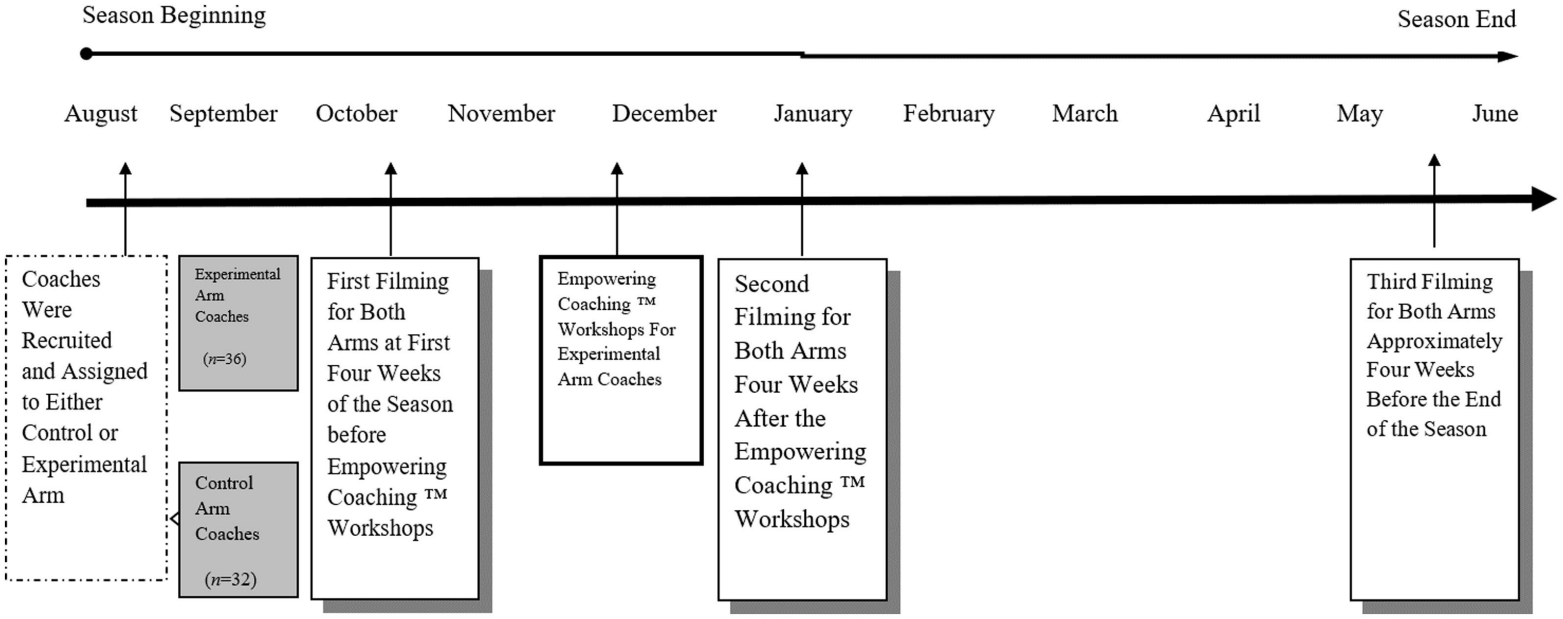
Timeline for delivery of the *Empowering Coaching*™ workshop and the three time points of data collection (filming) across a season.

This study adopted a longitudinal, quasi-experimental design with repeated measures. Filming was conducted three times across the course of an athletic season (Figure 2). Regarding each of the targeted time points, we considered (on average) how many times the coach would have the opportunity to interact with their players. In general, including training sessions (1–1.5 h in length) and matches (on average, 1.5 h in length), there were two to three interactions per week. As the sport literature suggests that it takes approximately 3–4 weeks before the coach-created motivational climate on a team is realized (e.g., Gano-Overway and Ewing, 2004), the first filming (T1; baseline) took place during the first 4 weeks of the season for both groups of coaches. This T1 filming occurred before intervention coaches participated in an approximately 6-h classroom-based version of the *Empowering Coaching*™ workshop.

The second filming (T2) of the intervention and control group coaches occurred approximately 1 month after the intervention coaches attended the *Empowering Coaching*™ workshop. It was assumed that this would allow time for the coaches to try to implement more empowering behaviors and reduce disempowering behaviors (a minimum of approximately eight training or match sessions). The third filming for both groups of coaches took place approximately 4–6 weeks before the end of the season to avoid overburdening coaches and teams at the busy phase of the season. The exact time of filming (i.e., particular day and week) depended on the availability of coaches.

Before the first filming day (T1), a researcher visited the training site to familiarize participants with the filming procedure and the presence of the camera (Van der Mars, 1989; Tessier et al., 2010). During the day of the filming, the researcher arrived before the start time to set up the video equipment (camera and tripod) and attached a wireless microphone to the coach. To minimize reactivity to the video cameras and prevent the presence of the camera from interfering with the training session, cameras were placed in an unobtrusive section of the play area (Tessier et al., 2013). After each filmed training session, the audio and video files were synchronized. The video files were then examined to ensure the audio and visual data were of acceptable quality for subsequent coding.

Coding was carried out using the MMCOS because it revealed satisfying psychometric properties, as partial least squares confirmatory analysis provided evidence of adequate factorial validity of the instrument, as well as evidence of predictive validity for lower-order environmental dimensions (Smith et al., 2015, 2016a,b). Coding of the video footage was conducted independently by two raters from each country who were blind to the specific aims of the study and were unaware of the classification of coaches in terms of whether they were in the intervention or control arm. Rigorous rater training procedures were followed to ensure inter- and intra-rater agreement (Smith et al., 2015).

Reliability for all codings across all countries was examined; all coefficients exceeded the cutoff value (*k* > 0.70). Raters were academic staff and doctoral students in the discipline of sports psychology. Each possessed a strong knowledge of the conceptual and theoretical background of the study, as well as experience in teaching and/or coaching football (Smith et al., 2015).

Since the duration of training sessions varied significantly, we adopted an approach for analyzing the footage that differed from methodologies utilized in previous systematic observations (e.g., Van der Mars, 1989). This analytical approach involved splitting videos into four quarters to ensure that all recorded footage was coded, as even very brief interactions occurring in-between time blocks may significantly affect the motivational atmosphere. Raters coded the footage according to a marking scheme that took into account the style and range of strategies employed by the coaches, as well as the impact (e.g., perceived intensity and individual or group effects) of strategies on the climate. At the end of each quarter, raters coded the potency of each of the seven environmental dimensions on a scale of 0 (*very low potency*) to 3 (*very strong potency*). Potency refers both to frequency (i.e., number of behavioral strategies used for each dimension during the coding interval) and the quality (i.e., pervasiveness and quality of coaches’ behaviors) of each strategy. After raters coded the entire training session, they provided an overall rating of the degree to which the coaching atmosphere was empowering and disempowering using the same scale (Smith et al., 2015).

### 2.3 Intervention workshops

Coaches in the intervention condition attended the approximately 6-h *Empowering Coaching*™ classroom-based training program (Duda, 2013). Coach educators—chosen because of their coaching experience and likelihood of delivering coach education courses within their football association—were trained by the respective research team in each country to deliver the *Empowering Coaching*™ workshops. These experienced coach educators delivered the *Empowering Coaching*™ training face-to-face to the intervention grassroots coaches in two approximately 3-h workshops carried out 1 week apart (or within a 6 + h workshop delivered in a day). The workshops took place in the football club of the grassroots coaches (e.g., a suitable meeting room), involving a group of 5 to 10 participants. The experienced coach educators volunteered to be trained up and to deliver the workshops, and did not receive any financial remuneration. The coach educators introduced the intervention grassroots coaches to the conceptual (and empirical) bases of an “empowering approach” to coaching and aimed to enhance their understanding of the processes involved in optimizing players’ autonomous motivation and optimal engagement in sport.

The *Empowering Coaching*™ training program comprises the following active ingredients to help grassroots coaches understand and apply the principles of the *Empowering Coaching*™ in their current coaching behaviors in trainings, matches, and across a football season: (1) interactive activities to allow coaches to identify what they expect and want to learn from this coaching training and describe their current coaching philosophy and then reflect on it; (2) an in-depth presentation of the key theoretical principles of *Empowering Coaching*™; (3) an interactive activity to help grassroots coaches apply these principles by categorizing several coaching behaviors as being “positive,” “negative,” or could be one or the other dependent on circumstances; (4) the viewing and analyzing, based on workshop content, video clips illustrating *Empowering Coaching*™ behaviors/strategies; (5) planning the next training sessions with an aim to having those sessions be more empowering; and (6) identifying the obstacles to implementing the *Empowering Coaching*™ behaviors and proposing solutions to overcome these obstacles.

The intervention prototype was developed by the University of Birmingham based on Duda’s (2013) research and applied work in this area. The materials produced were culturally adapted and translated into the languages of the project partners, and an intervention prototype was then piloted in each participating country. The *Empowering Coaching*™ intervention package, as implemented within the PAPA project, involved audiovisual materials (PowerPoint presentations, subtitled DVDs) and workbooks to summarize and aid retention of key content. In addition, a website was developed in each country (i.e., www.empoweringcoaching.co.uk) to support the implementation of the intervention across the season. A detailed description of the conceptual and empirical foundations of the program, as well as the key features of the intervention, can be found elsewhere (see Duda, 2013; Duda et al., 2013). The details of the intervention are also summarized in a TIDieR checklist (see Table 1).

**Table 1:** Coach demographics by condition.

Participant coaches by condition	Intervention group coaches (*n* = 36)	Control group coaches (*n* = 32)
Mean age (years)	35.2 (SD = 9.3)	36.1 (SD = 10.1)
Coaching experience (years)	6.7 (SD = 3.7)	7.2 (SD = 4.9)
Countries represented	UK, France, Greece, Spain	UK, France, Greece, Spain

### 2.4 Statistical analyses

To test our hypotheses, multilevel analyses were performed using SPSS MIXED MODELS (v22). Within the framework of multilevel modeling, repeated measurements in longitudinal design studies are treated as nested data, where multiple observations are nested within individual participants (Peugh, 2010). To examine the major research questions and following the recommendations of Singer and Willett (2003), an unconditional model (Model 1)—with only an intercept and no explanatory variables—was tested in a preliminary step. This model partitions the variance of each dependent variable into within-individual and between-individual components and permits the examination of the intra-class correlation. In a second step, an unconditional linear growth curve model (Model 2) was tested by including the variable “time” as a fixed parameter. This variable represented the linear change in the treatment condition over time. In Step 3, the variable “condition” (a dummy variable in which the treatment condition = 1 and the standard condition = 0) and the interaction term “Time × Condition” were added as predictors (Model 3). The “condition” effect tested whether the treatment condition and the standard condition differed at baseline (time = 1), while the interaction “Time × Condition” examined whether the linear rate of change over time differed across conditions. Finally, in Step 4, quadratic parameters (i.e., time by time and time by time by group) were included in the models. This allows detection of whether there are any changes in the rate of change (acceleration or deceleration) over the three measurement occasions (Heck et al., 2010).

Models were compared based on the −2 log likelihood (i.e., likelihood ratio test/deviance test), with lower values indicating better model fit. Due to space restrictions, only the results of Models 3 and 4 are presented. An estimate of the effect size was reported using *R*^2^ conditional (i.e., provides the variance explained by the entire model) and *R*^2^ marginal (i.e., provides the variance explained only by fixed effects). All data presented in this study are available upon request from the corresponding author.

## 3 Results

### 3.1 Descriptive statistics

Descriptive statistics in terms of mean values for each variable for both groups at each measurement occasion are provided in Table 2.

**Table 2:** Descriptive statistics of motivational climate dimensions for all 3 measurement time points.

Environmental climate dimensions	AS	CO	TI	EI	RS	RT	ST	EMP	DIS
Mean experimental^a^	1.05	1.06	1.50	0.43	1.46	0.58	1.84	1.63	1.03
Mean control^a^	0.83	1.22	1.47	0.51	1.28	0.69	1.82	1.45	1.21
SD experimental^a^	0.64	0.53	0.56	0.42	0.62	0.55	0.47	0.67	0.56
SD control^a^	0.72	0.56	0.64	0.44	0.70	0.59	0.55	0.72	0.66

Experimental group *n* = 36; Control group *n* = 32. AS, autonomy support; CO, controlling; TI, task-involving; EI, ego-involving; RS, relatedness support; RT, relatedness thwarting; ST, structured; EMP, empowering; DIS, disempowering.

a Numbers are derived from a 3-point rating scale used by trained raters.

### 3.2 Main analyses

**Figure 3:**
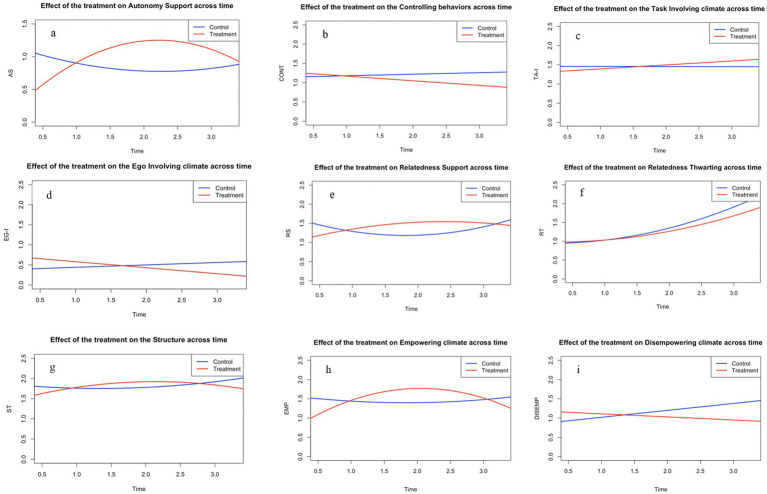
Graphic representations of the objective motivational climate dimensions including autonomy support **(a)**, controlling **(b)**, task-involving **(c)**, ego-involving **(d)**, relatedness support **(e)**, relatedness thwarting **(f)**, structured **(g)**, empowering **(h)**, and disempowering **(i)**, broken down by experimental condition and measurement occasion. Time 1 = Time Point 1 (pre-intervention); Time 2 = Time Point 2 (post-intervention); Time 3 = Time Point 3 (post-intervention); numbers are derived from a 3-point rating scale used by trained raters. Blue lines represent the control group, while red lines represent the experimental group.

Tables 3, 4 present the results of the multilevel growth models. Graphic representations of the objective climate dimensions broken down by experimental condition and measurement occasion are illustrated in Figure 3.

**Table 3:** Parameter estimates for linear and quadratic multilevel models examining the effectiveness of the *Empowering Coaching*™ program across the two higher order factors.

Environmental dimensions	Empowering (model 4)	Disempowering (model 3)
	Estimate (SE)	Estimate (SE)
Fixed effects		
Intercept	1.60*** (0.40)	0.84*** (0.15)
Time	−0.22 (0.44)	0.18 (0.06)
Condition	−1.01 (0.54)	0.35 (0.21)
Time*Condition	1.37* (0.61)	−0.26*** (0.09)
Time*Time	0.06 (0.11)	
Time*Time*Condition	−0.34* (0.15)	
Random effects		
W. C. V.	0.25*** (0.03)	0.27*** (0.03)
B. C. V.	0.22*** (0.05)	0.08* (0.03)
ICC	0.49	0.24
R2 marginal	0.02	0.06
R2 conditional	0.49	0.29
Model fit		
*−*2 LL (Unconditional)	373.06	343.21
*−*2 LL (model 3)	362.35	326.30
*−*2 LL (model 4)	355.16	323.70

Bold indicates best fitting model with respective confidence intervals. W.C.V, within-coach variance; B.C.V, between-coach variance; I.C.C., intraclass correlation coefficient; SE, standard error; LL, log likelihood; ^a^ 0 = control group coaches, 1 = intervention group coaches; reference category = control group coaches. ^†^*p* < 0.10. *p < 0.05. **p < 0.01. ***p < 0.001.

**Table 4:** Parameter estimates for linear and quadratic multilevel models examining the effectiveness of the empowering coaching™ program across the selected environmental dimensions.

*Environmental dimensions*	Autonomy supportive (model 4)	Controlling (model 3)	Task-involving (model 3)	Ego-involving (model 3)	Relatedness supportive (model 4)	Relatedness thwarting (model 4)	Structure (model 4)
	Estimate (SE)	Estimate (SE)	Estimate (SE)	Estimate (SE)	Estimate (SE)	Estimate (SE)	Estimate (SE)
Fixed effects							
Intercept	1.18*** (0.36)	1.14*** (0.13)	1.46*** (0.12)	0.38*** (0.10)	1.71*** (0.34)	0.95* (0.40)	1.86*** (0.27)
Time	−0.36 (0.40)	0.04* (0.06)	−0.003* (0.04)	0.06 (0.04)	−0.58 (0.38)	−0.04 (0.46)	−0.16 (0.30)
Condition	−1.06* (0.49)	0.15 (0.18)	−0.17 (0.18)	0.35* (0.15)	−0.74 (0.47)	0.03 (0.56)	−0.44 (0.37)
Time*Condition	1.38* (0.54)	−0.16^†^ (0.08)	0.10 (0.06)	−0.21** (0.06)	1.06* (0.52)	0.002 (0.63)	0.63 (0.41)
Time*Time	0.08 (0.09)				0.16 (0.10)	0.12 (0.12)	0.06 (0.08)
Time*Time*Condition	−0.31* (0.13)				−0.26* (0.13)	−0.03 (0.15)	−0.17 (0.10)
Random effects							
W. C. V.	0.19*** (0.02)	0.21*** (0.02)	0.12*** (0.01)	0.13*** (0.01)	0.18*** (0.02)	0.18*** (0.02)	0.11*** (0.01)
B. C. V.	0.24*** (0.05)	0.07**(0.02)	0.23***(0.05)	0.04*** (0.01)	0.24*** (0.05)	0.24*** (0.05)	0.14*** (0.03)
*ICC*	0.56	0.25	0.66	0.24	0.58	0.16	0.57
R2 marginal	0.05	0.05	0.01	0.06	0.02	0.02	0.07
R2 conditional	0.58	0.30	0.67	0.28	0.58	0.18	0.57
Model fit							
−2 LL (Unconditional)	353.84	307.61	274.91	250.54	332.50	329.14	237.06
−2 LL (model 3)	335.10	292.70	263.74	201.04	321.47	318.15	230.07
−2 LL (model 4)	328.27	288.92	260.98	260.60	317.35	316.44	227.04

Bold indicates best fitting model with respective confidence intervals. W.C.V, within-coach variance; B.C.V, between-coach variance; I.C.C., intraclass correlation coefficient; SE, standard error; LL, log likelihood; ^a^ 0 = control group coaches, 1 = intervention group coaches; reference category = control group coaches; 95% CI, 95% confidence intervals. ^†^*p* < 0.10. *p < 0.05. **p < 0.01. ***p < 0.001.

For the higher order factors (see Table 3), results concerning empowering climate show that Model 4 (i.e., with quadratic parameters) is the best one, in which both Time × Condition (*b* = 1.37, *p* < 0.05) and Time × Time × Condition (*b* = −0.34, *p* < 0.05) interaction effects are significant. Visual inspection of Figure 3h suggests that intervention coaches showed a temporal increase in observed overall empowering climate behaviors from T1 to T2, but a decrease in these behaviors from T2 to T3. Fixed parameters (marginal *R*^2^) explained 2% of the total variance, while both fixed and random parameters (conditional *R*^2^) explained 49% of the total variance of teachers’ empowering coaching behaviors.

Pertaining to observed disempowering climate behaviors, results revealed that Model 3 is the best one, with a significant effect of the interaction Time × Condition (*b* = −0.26, *p* < 0.001). This suggests that after the intervention, there was a significant linear decline in the overall disempowering climate created by intervention coaches on their teams over the course of the football season (see Figure 3i). Fixed parameters (marginal *R*^2^) explained 6% of the total variance, while both fixed and random parameters (conditional *R*^2^) explained 29% of the total variance in disempowering coaching behaviors.

As for the empowering climate sub-dimensions (see Table 4), results show that Model 4 is the most appropriate model for autonomy support, relatedness support, and structure. More specifically, for autonomy support, the Time × Condition (*b* = 1.38, *p* < 0.05) and Time × Time × Condition (*b* = −0.31, *p* < 0.05) interaction effects were significant. For relatedness support, both Time × Condition (*b* = 1.06, *p* < 0.05) and Time × Time × Condition (*b* = −0.26, *p* < 0.05) interaction effects were significant. Regarding structure, however, the Time × Condition (*b* = 0.63, *p* = 0.13) and Time × Time × Condition (*b* = −0.17, *p* = 0.11) interaction effects were not significant. Finally, for observed task-involving behaviors, results revealed that Model 3 is the more appropriate model and that the Time × Condition (*b* = 0.10, *p* = 0.10) interaction effect was not significant. Considering Figures 3a,c,e,g, the latter findings imply that coaches from the intervention group increased their autonomy support and relatedness support from T1 to T2, but were observed to decrease these behaviors from T2 to T3. Furthermore, the findings indicate that the intervention coaches did not improve the provision of structure or the exhibiting of task-involving behaviors. Fixed parameters explained 5 and 2% of the total variance of autonomy support and relatedness support, respectively, while both fixed and random parameters explained 58% of the total variance in ratings of these sub-dimensions.

Finally, with respect to the disempowering climate sub-dimensions (see Table 4), results show that Model 3 is the most appropriate model for controlling behaviors and ego-involving climate, while Model 4 is more appropriate for relatedness-thwarting. More specifically, the Time × Condition interaction effect approach significance for controlling behaviors (*b* = −0.16, *p* = 0.06) but was significant for ego-involving climate (*b* = −0.21, *p* < 0.01). As for relatedness-thwarting, results show that both interaction effects, Time × Condition (*b* = 0.002, *p* = 0.99) and Time × Time × Condition (*b* = −0.03, *p* = 0.85), were not significant. Visual inspection of Figures 3b,d,f suggests a decrease in controlling and ego-involving behaviors over time and a non-significant change in relatedness thwarting. Fixed parameters explained 5 and 6% of the total variance of controlling behaviors and ego-involving climate, respectively, while both fixed and random parameters explained 30 and 28% of the total variance in these variables.

## 4 Discussion

This study assessed the effectiveness of the *Empowering Coaching*™ (Duda, 2013) coach education program on the observed motivational climate in youth football across four European countries (i.e., England, France, Spain, and Greece). Participation in the *Empowering Coaching™* program significantly improved the motivational climate created by coaches over the course of the season (on five of the nine variables assessed, capturing characteristics of that climate), compared to coaches in the control group who did not receive the workshop. In particular, the *Empowering Coaching*™ program produced a sustained decrease (i.e., linear effect) in the *disempowering* dimensions of the coach-created motivational climate (i.e., overall disempowering environment and ego-involving features of the climate), and produced short-term, but not sustained (i.e., quadratic effect), improvements in the empowering dimensions assessed (i.e., the overall empowering environment, autonomy-support, and relatedness support features of the climate). Approaching statistical significance, participation in the *Empowering Coaching™* training corresponded to a linear decrease in the exhibited controlling behaviors of the intervention coaches across the three time points.

In line with the results from other interventions in sport (e.g., Smith et al., 2007) and PE settings (e.g., Aelterman et al., 2014; Espinoza-Gutiérrez et al., 2024), our findings indicate that coach in-service training can promote a coach-created motivational atmosphere marked by enhanced need-supportive features and decreased need-thwarting strategies. The present results suggest that the *Empowering Coaching*™ approach to coach training was effective, as coaches in the intervention arm appeared to create a more adaptive motivational environment in their teams.

We assume that these effects are probably due to the core principles of the intervention; participant coaches were not asked to modify their coaching philosophies and approaches, but rather they were trained to recognize and understand the implications of both adaptive and maladaptive coaching practices and motivational processes. As a result of this understanding, the overarching aim of the training was to have the workshop participants “internalize the messages conveyed and feel more committed and competent about becoming more empowering coaches” (Duda and Appleton, 2016, p. 373). *Empowering Coaching*™ training content and embedded learning activities might have also made coaches more aware and probably more “sensitive” to the maladaptive effects and consequences of disempowering strategies on youth sport participants. Through workshop activities, including discussions with fellow coaches, they had the opportunity to consider the behaviors they wanted to modify and to generate alternative coaching behaviors—more empowering strategies—to minimize disempowering practices on the pitch.

Sustained improvements (across the season) were most evident in the reduction of ego-involving behaviors in the intervention group. This finding suggests that coaches were especially responsive to training content that addressed the potential harm of ability-based comparisons and emphasized the importance of demonstrative (relative to others) superior competence. As such, we would expect the observed changes in the motivational climate to be particularly beneficial (or at least, less detrimental) for young players who have doubts about their competence. Exerted effort, degree of engagement, skill development, and performance are expected to be compromised when perceptions of one’s ability are low and the climate is ego-involving (Duda, 2001; Nicholls, 1989). Recent research (Espinoza-Gutiérrez et al., 2024) also points to the detrimental effects of ego-involving climates on athlete wellbeing. As such, the present findings suggest that the *Empowering Coaching*™ training would hold positive implications for athletes’ psychological welfare.

Short-term improvements (from baseline to approximately 1 month following the workshop) were observed in autonomy-supportive and relatedness-supportive practices. Coaches in the intervention arm following the training appeared to have valued and incorporated strategies to promote players’ feelings of autonomy (e.g., providing meaningful choices) and relatedness (e.g., demonstrating that the coach cares about the young player beyond their play on the “pitch”) when interacting with their teams. However, it seems that after the initial classroom-based workshop, the coaches tended to revert to their usual motivational practices (regarding supporting player autonomy and relatedness), either because of the lack of effective follow-up training activities or due to external pressures and constraints (Mageau and Vallerand, 2003), such as season-related competitive demands. Based on the present findings, it appears that a “proximity effect” took place as coaches had a vivid training experience during the first few weeks or months after the intervention, but those particular effects began to wear off as the season progressed. This finding aligns with past intervention studies advocating the need for top-up sessions or follow-up support during the season (García-Cazorla et al., 2024) to maintain adaptive behaviors.

Although only a trend, results revealed a hypothesized decrease in coach controlling behaviors. Taking into consideration that the baseline measurement scores suggested a relatively low level of controlling behaviors before the intervention, it might have been challenging for the intervention coaches to substantially further modify a motivational environment already low in controlling features.

Regarding the task-involving structure and relatedness-thwarting aspects of the observed coach-created motivational climate, the effects of the intervention were non-significant. This might be due to coaches’ greater focus on exhibiting, in grassroots sport, behaviors that provide structure and emphasize that young players should improve and master their skills, try hard, and do their best. Similarly, particularly at the youth sport level, we would expect (and hope that this is the case) that coaches’ thwarting of players’ feelings of relatedness would be rare. Current findings (when examining observed Time 1 mean values) are aligned with this assumption and aspiration (see Table 2).

Several reasons might explain the lack of some significant effects or the lack of large effects of the intervention on coaching behaviors. One of them is pressure, from the club and from parents, toward grassroots coaches to have their teams perform successfully and maximize the team’s win–loss record. Having in mind that in some countries (e.g., Greece), grassroots coaching is largely a paid profession, in many cases, parents and clubs compel coaches to emphasize competitive outcomes. Such pressures and contingencies may influence the coaches themselves. Past research has indicated detrimental effects of club pressure on coach motivation and psychological wellbeing (Alcaraz et al., 2015). Research has also indicated that less intrinsically motivated coaches are more reluctant to implement coaching innovations (e.g., Gorozidis and Papaioannou, 2014).

A final suggestion deriving from the present findings is that, although not possible in the PAPA project, future attempts to implement *Empowering Coaching*™ should consider the need for “top-up” training for coaches as the season progresses. Indeed, these results underscore the importance of ongoing, context-specific support for coaches. One-off (and in the case of the PAPA project, only classroom-based) workshops may create the desire to potentially initiate change, but sustained impact likely requires follow-up, mentoring, practical (“on the pitch”) training, and/or digital resources that reinforce learning throughout the season. The current findings are in line with past studies (e.g., Cheon et al., 2012), implying that supplementary training across the season might strengthen the effects of the intervention and produce an even more stable, more empowering (and less disempowering) coach-initiated team climate.

## 5 Limitations

The present study does not come without certain limitations. The present report draws conclusions from data deriving from a single source, that is, observational data. Furthermore, only 2 of 68 coaches were female, limiting the generalizability of the present findings. In terms of study design, although relatively strong for a longitudinal design (three time points over the course of a season), nine coaches were lost to follow-up. Finally, the fidelity of intervention delivery across countries was not formally assessed to allow determination of whether the level of fidelity moderated the observed effects.

## 6 Conclusion

This study provides empirical support for the *Empowering Coaching*™ program as an effective strategy to enhance the motivational climate in grassroots football. Coaches who received the *Empowering Coaching*™ training demonstrated sustained reductions in disempowering behaviors, particularly ego-involving practices, and short-term improvements in autonomy and relatedness support. The results also support the intervention’s cross-cultural applicability and its potential to inform coach education policies that prioritize youth wellbeing and sustained sport participation. The findings suggest that while an approximately 6-h classroom-based intervention session can initiate behavior change, ongoing support may be necessary to maintain empowering coaching practices across a season. Future work should explore integrating continuous learning opportunities, assessing athlete outcomes, and examining coach behaviors using a multi-method approach (e.g., observation and questionnaire) in training and competition (Smith et al., 2016a,b) across diverse sporting environments, including individual sports and at more elite levels.

## Data Availability

The raw data supporting the conclusions of this article will be made available by the authors, without undue reservation.
